# Pediatric Ménétrier's Disease Triggered by *Cytomegalovirus* Infection: A Rare Case of Severe Hypoalbuminemia and Edema

**DOI:** 10.1155/crpe/2471369

**Published:** 2025-10-31

**Authors:** Issa Snoubar, Tariq Alhendi, Dalia Batanji, Noor Nabresi, Mutaz Sultan, Sami Bannoura, Kamaledein Einar

**Affiliations:** ^1^Department of Medicine, An-Najah National University, Nablus, State of Palestine; ^2^Department of Pediatrics, Al-Makassed Islamic Charitable Society Hospital, Jerusalem, State of Palestine; ^3^Department of Pediatrics, Dr. Thabet Thabet Hospital, Tulkarem, State of Palestine; ^4^Department of Pediatrics, Pediatric Gastroenterology, Al-Makassed Islamic Charitable Society Hospital, Jerusalem, State of Palestine; ^5^Department of Pathology, Al-Makassed Islamic Charitable Society Hospital, Jerusalem, State of Palestine; ^6^Department of Lymphoma/Myeloma, Anderson Cancer Center, Houston, Texas 77030, USA

## Abstract

**Introduction:**

Ménétrier's disease (MD) is a rare protein-losing gastropathy characterized by hypertrophy of the gastric mucosa, particularly in the fundus and body, leading to hypoalbuminemia and peripheral edema. The etiology remains unclear, but in children, MD is frequently associated with *Cytomegalovirus* (CMV) infection. Pediatric cases usually have an acute, self-limiting course and may respond to supportive care.

**Case Presentation:**

We present a case of a 3-year-old previously healthy male who developed vomiting, fatigue, and progressive edema, including facial, limb, and testicular swelling. Laboratory evaluation revealed significant hypoalbuminemia (2.3 g/dL), hypoproteinemia, hypogammaglobulinemia, elevated triglycerides, and lymphocytosis. Abdominal ultrasound showed free fluid and dilated bowel loops. Esophagogastroduodenoscopy revealed thickened gastric folds, and biopsy confirmed MD with CMV inclusion bodies and positive CMV immunostaining, despite negative CMV PCR and serology. The patient received supportive treatment including albumin infusions, diuretics, high-protein diet, and IV ganciclovir, with clinical improvement.

**Discussion:**

This case reinforces the known association between CMV and MD in children and highlights the variability of CMV detection via PCR or serology. Although pediatric MD is often self-limiting, antiviral therapy may be indicated in severe cases or when supportive care is insufficient. The presence of hypogammaglobulinemia in this case also raises questions about potential underlying immunodeficiency. Clinicians should maintain a high index of suspicion for CMV-associated MD in children with acute-onset edema and hypoalbuminemia, even when routine viral testing is negative.

## 1. Introduction

Ménétrier's disease (MD) is a rare disorder, considered as a part of protein-losing gastropathy in adults and children; it is characterized by gastric hypertrophy, especially in the fundus and body due to gastric mucosal thickening [1]. The disease is also associated with hypoalbuminemia due to leakage of albumin into the gastric lumen [2]. The exact cause of MD remains unclear [3], but several cases have described a possible association between MD and *Cytomegalovirus* (CMV) infections, especially in children [4].

The disease presentation differs according to age group [1]. Children's presentations are usually acute and are characterized by abdominal manifestations such as abdominal pain, vomiting, and diarrhea. Patients also present with peripheral edema and even anasarca [5]. In contrast, adults present with epigastric pain, diarrhea, edema, weight loss, and anemia from gastric bleeding due to erosions [6].

The diagnosis of MD is mainly clinical; however, investigation could be used to verify the diagnosis such as esophagogastroduodenoscopy (EGD), abdominal ultrasound (US), gastric biopsy, and other modalities such as chest US or radiography [4]. Laboratory findings may include hypoalbuminemia, hypoproteinemia, eosinophilia, and elevated levels of fecal *α*1-antitrypsin [4].

The management and course of disease are benign in children and resolve spontaneously in 4–6 weeks or require only supportive care such as a high-protein diet, albumin infusion, proton pump inhibitors (PPIs), and diuretics according to clinical manifestation [7]. In contrast, this condition in adults is more progressive and requires more follow-up [6].

In this case, we present a 3-year-old male patient with severe symptoms due to hypoalbuminemia, such as testicular swelling and frequent albumin infusion. The disease was associated with CMV infection.

## 2. Case Presentation

A 3-year-old male patient who was in his usual state of health until 2 weeks ago complained of fatigue and vomiting that was nonbilious and nonbloody. After 4 days of vomiting, the patient had facial swelling mainly in the periorbital region. The swelling generalized to include bilateral lower limbs and testicular area. The patient has a history of gastroesophageal reflux disease (GERD) during infancy and has experienced recurrent nocturnal vomiting every 2-3 days over the past year. The episodes increased in severity over the past 2 weeks but resolved following initiation of a PPI. His family history is remarkable for familial Mediterranean fever (FMF) that occurred in his mother and sister confirmed by genetic testing, but the patient himself has not ever been tested for FMF. His developmental history is normal up to his age. He can alternate feet going up the stairs and copy a circle. He knows his age and gender and can speak 3-word sentences.

On exam at admission, his weight was 15.2 kg (68.082 percentile) while his height was 99 cm (82.639 percentile). He had periorbital edema without icteric sclera, distended abdomen, bilateral lower limb pitting edema extending to the knees, edema of both hands, nonpitting sacral edema, and bilateral marked testicular swelling. No hepatosplenomegaly was noted, and the abdomen was tympanic on percussion.

Initial labs showed that total protein was low (3.4 g/dL), albumin was low (2.3 g/dL), IgM, IgE, IgG, and IgA were low (38, 27, 96, and 31 mg/dL, respectively), and triglycerides were high (737.5 mg/dL). No other immunological diagnostic workup has been performed. CBC showed increased white blood cell count (13,600 cells/μL) with increased percentage of lymphocytes (63.2%) and eosinophiles (0.831%) while decreased neutrophils (24.4%), low hemoglobin (11.7 g/dL) and MCV (68.7 FL), and increased platelets (558,000 platelets/μL).

Hepatic enzymes, panel for HCV and HBV, and bilirubin were normal. His blood urea nitrogen was normal (17.7 mg/dL) with low creatinine (0.17 mg/dL). ESR and electrolytes were normal. 24 urine collection and urine analysis were unremarkable and without proteinuria. Also, calcium to creatinine ratio and protein to creatinine ratio were normal. However, a fecal sample to quantify alpha1-antitrypsin level has not been collected.

**Figure 1:**
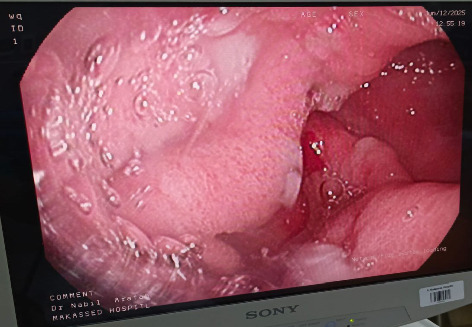
Upper gastrointestinal endoscopy demonstrating markedly hypertrophied gastric folds with mucosal thickening, consistent with hypertrophic gastropathy.

Abdominal US was done to the patient and showed mildly dilated bowel loops, small to moderate volume of intraperitoneal free fluid in the abdomen and pelvic cavities with few septations, and no hepatosplenomegaly. Thus, EGD revealed congested gastric body and fundus with thickened gastric folds and mucosal hypertrophy that was suggestive of hypertrophic gastropathy (Figure 1). Otherwise, the esophagus, antrum, and the first and second portions of the duodenum looked normal.

**Figure 2:**
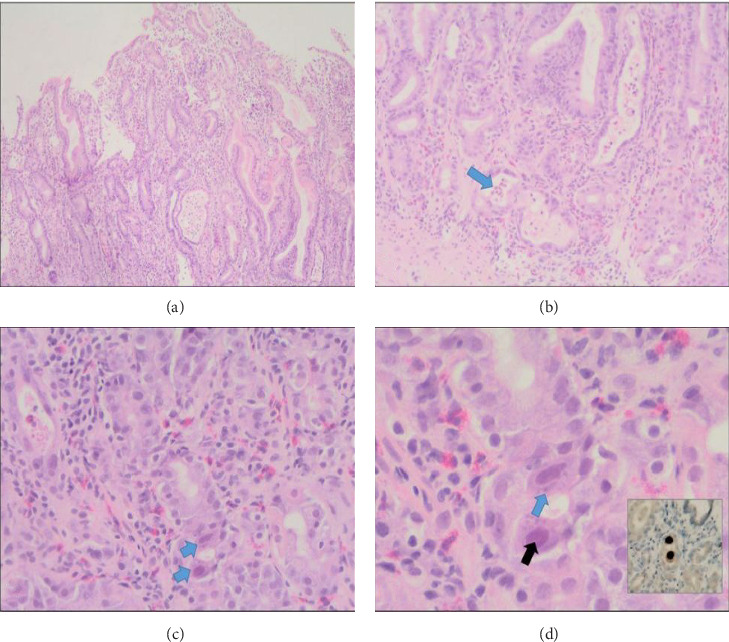
Ménétrier's disease with associated CMV viral cytopathic changes. (a) Sections show corpus-type gastric mucosa with prominent hyperplastic changes (H&E; 4X). (b) Moderate active inflammation with dilated gastric pits and pit abscess (arrow) is noted (H&E; 10X). (c) Presence of viral cytopathic changes characteristic of *Cytomegalovirus* (H&E; 20X). (d) Higher power view shows the characteristic eosinophilic intranuclear inclusion (black arrow) as well as the cytoplasmic basophilic inclusions (blue arrow). The insert shows positive CMV immunostain in the same cells.

Histopathological examination of gastric biopsies demonstrated moderate active gastritis with mucosal hyperplastic changes and CMV cytopathic features with positive immunostain and inclusion bodies present (Figure 2). However, CMV serologic testing showed that IgG and IgM were within normal ranges. In addition, CMV DNA polymerase chain reaction (PCR) was negative 1 week after admission, while the duodenal biopsies were with no pathologic abnormalities.

Management included multiple intravenous albumin infusions, intermittent furosemide, maintained on high-protein and low-salt diet with medium-chain triglyceride (MCT) oil supplementation, esomeprazole, and IV ganciclovir for 5 days; then, the infectious disease specialist recommended to stop it. This led to clinical improvement with albumin rising to 3.17 g/dL during last days of admission.

At discharge ten days later, the patient was hemodynamically stable and active, with improvement of anasarca on physical exam. Then, follow-up albumin levels were measured twice, with results of 2.8 g/dL and 3.3 g/dL, respectively. Follow-up triglyceride level was trending down but still high (277 mg/dL), while follow-up immunoglobulin was not done.

## 3. Discussion

MD is a part of protein-losing gastropathy in adults and children. The first was described by Pierre Ménétrier and reported in 1888 [1]. It is characterized by hypertrophy of the gastric mucosa in the body of the stomach and foveolar hyperplasia, hypoalbuminemia, and resulting peripheral edema [1].

The exact cause of the disease was not fully understood; it could be allergic, immunologic, or infectious such as CMV and *Helicobacter pylori* (*H. pylori*) [8]. Other infectious diseases such as herpes simplex, *Mycoplasma*, and *Giardia lamblia* have been implicated [2].

There are several case reports that show an association between CMV and MD, especially in children. On the other hand, in adults, there is more association with *H. pylori* infection [6]. Although the association between CMV and MD is reported in up to 70% of pediatric cases with MD [9], the pathogenesis of how CMV affects the gastric mucosa is not fully understood.

Few studies have described the association: Transforming growth factor alpha (TGF-α) which is elevated in pediatric MD binds to epidermal growth factor receptor (EGFR) on the mucosa of the stomach, leading to foveolar hyperplasia, hypertrophy in the body and fundus, decrease in acidic secretion, proliferation of mucus-secreting cells, and increase in probability of neoplastic transformation [10]. The infection with CMV may lead to an increased production of TGF-α [4].

Another important event that occurs in MD is hypoalbuminemia, which occurs through increased vascular permeability and leakage of protein from wider tight junctions of gastric mucosa cells [11].

The manifestation and course of the disease vary according to the patient's age. The clinical presentation of the disease in children is characterized by an acute onset of edema due to hypoalbuminemia, associated with general manifestations such as abdominal pain, diarrhea, and vomiting, as in our case. More symptoms have been reported rarely, such as fever, hepatosplenomegaly, and rash [4]. In contrast, adults present with epigastric pain, diarrhea, edema, weight loss, and anemia from gastric bleeding due to erosions [6].

The diagnosis of MD is mainly clinical. However, other investigations could aid in diagnosis, such as hypoalbuminemia and hypoproteinemia in the majority of patients, peripheral eosinophilia in 25% of patients, and an increased level of fecal *α*1-antitrypsin in 33.3% of cases [4]. The presence of hypoproteinemia may warrant a biopsy, which typically reveals glandular atrophy and inflammatory infiltration [5].

Other investigations, such as EGD, may be used. EGD is considered a gold standard for diagnosis, which reveals hypertrophic gastric folds and foveolar hyperplasia [12]. An abdominal US is used to confirm the diagnosis by detecting hypertrophic gastric folds and thickening of the gastric wall. Other modalities, such as chest US or radiography, can detect pleural effusion [4].

It is important to assess CMV in any case of pediatric MD. Initially, it can be screened using serologic testing for anti-CMV IgM antibodies. Detection of inclusion bodies can support the diagnosis, although they are not always present. However, using PCR to detect CMV DNA in the gastric biopsy is the most sensitive test for confirmation of the diagnosis [4, 13].

However, in our case, the patient had a negative PCR test with a biopsy result showing typical CMV changes; this incident might be due to localized inflammation in our case; it might also be due to lab error, or it could implicate our knowledge about the sensitivity of such tests in MD. This finding can be assessed by monitoring other cases to validate whether these findings are solitary in our case or if studies should assess the validity of these labs in MD patients specifically.

The course of MD in pediatrics is usually benign and resolves spontaneously within 4–6 weeks. Supportive care for patients may include pain controllers, a high-protein diet, PPIs, intravenous albumin infusions, diuretics, antacids, antiemetics, and steroids, according to clinical manifestation [2]. In our case, the patient required repeated albumin infusion, which reflected the severity of hypoalbuminemia.

Although it is rare to use antiviral therapy, there is a study suggesting to use ganciclovir treatment in some situations, such as infection in immunocompromised patients, neonates who have an immature immune system, and healthy infants who do not improve with supportive treatment after 2 weeks [13]. In our case, the patient required a course of ganciclovir due to lack of improvement after initial supportive management.

In addition, the unexplained hypogammaglobulinemia (low IgG and IgM) raises concerns for underlying immunodeficiency.

## 4. Conclusion

MD should be considered in children with unexplained hypoalbuminemia and generalized edema after excluding hepatic and renal causes. This case underscores the possible role of CMV, even in the absence of positive PCR or serology. Histopathology remains essential for diagnosis, and antiviral therapy may be beneficial in severe or nonresolving cases.
